# Supplementary material for: Efficacy of antimalarial drugs for treatment of uncomplicated falciparum malaria in Asian region: A network meta-analysis
Source: PLoS One. 2019 Dec 19;14(12):e0225882. doi: 10.1371/journal.pone.0225882 (PMC6922314; doi:10.1371/journal.pone.0225882)
Supplement: S2 Table — (PDF) [file pone.0225882.s002.pdf]

S2 Table. Search Terms

#13640

((((randomized controlled trial[pt] OR controlled clinical trial[pt] OR randomized[tiab] OR placebo[tiab] OR "drug therapy"[Subheading] OR randomly[tiab] OR trial[tiab] OR groups[tiab] NOT ("animals"[MeSH Terms] NOT "humans"[MeSH Terms])) AND ("malaria"[MeSH Terms] OR "malaria"[All Fields])) OR falciparum[All Fields]) AND ("antimalarials"[Pharmacological Action] OR "antimalarials"[MeSH Terms] OR "antimalarials"[All Fields])) NOT ("europe"[MeSH Terms] OR "europe"[All Fields])) NOT ("africa"[MeSH Terms] OR "africa"[All Fields])
