# Supplementary material for: Efficacy of antimalarial drugs for treatment of uncomplicated falciparum malaria in Asian region: A network meta-analysis
Source: PLoS One. 2019 Dec 19;14(12):e0225882. doi: 10.1371/journal.pone.0225882 (PMC6922314; doi:10.1371/journal.pone.0225882)
Supplement: S3 Table — (PDF) [file pone.0225882.s003.pdf]

S3 Table. Excluded studies and reasons for exclusion

	Study	Main reason
1	Dondorp et al 2009.<br>.	At 63 day follow- up
2	Satimai, 2012	Not an RCT
3	Tarning, et al 2008	Part of an included RCT
4	Grigg et al 2018	No comparator drug
5	Amaratunga et al 2016	Cohort design
6	Smithuis et al 2010	Day 63
7	Sirima et al. 2016	Not in Asia
8	Myint et al. 2017	One arm study
9	Tarning et al 2008.	Not address efficacy
10	Tjitra et al, 2012	Not at day 28
11	Kyaw et al 2013	Not RCT
12	Dondorp et al 2005	Difficult to extract 28 day outcome data
13	Wattanakoon, 2003	One arm study
14	Ghimire et al ,2018	One arm study
15	Wijeyaratne et al 2005	No comparator
16	Haque et al 2007	Not at day 28
17	Phong et al, 2019	Not at day 28

References

1. Dondorp AM, Nosten F, Yi P, Das D, Phyo AP, Tarning J, et al. 2009. Artemisinin resistance in *Plasmodium falciparum* malaria. N Engl J Med 361:455–467.

2. Satimai W, Sudathip P, Vijaykadga S, Khamsiriwatchara A, Sawang S, Potithavoranan T, Sangvichean A, Delacollette C, Singhasivanon P, Kaewkungwal J,

Lawpoolsri S. Artemisinin resistance containment project in Thailand. II: Responses to mefloquine-artesunate combination therapy among falciparum malaria patients in provinces bordering Cambodia. Malar J. 2012;11(1):300.

3. Tarning J, Ashley EA, Lindegardh N, Stepniewska K, Phaiphun L, Day NP, et al. Population pharmacokinetics of piperaquine after two different treatment regimens with dihydroartemisinin-piperaquine in patients with *Plasmodium falciparum* malaria in Thailand. Antimicrobial Agents and Chemotherapy 2008;**52**(3):1052–61.

4. Grigg MJ, William T, Piera KA, Rajahram GS, Jelip J, Aziz A, et al *Plasmodium falciparum* artemisinin resistance monitoring in Sabah, Malaysia: in vivo therapeutic efficacy and kelch13 molecular marker surveillance. Malar J. 2018;17(1):463.

5. Amaratunga C, Lim P, Suon S, Sreng S, Mao S, Sopha C, Sam B, et al. Dihydroartemisinin-piperaquine resistance in Plasmodium falciparum malaria in Cambodia: a multisite prospective cohort study. Lancet Infect Dis. 2016;16(3):357-65.

6. Smithuis F, Kyaw MK, Phe O, Win T, Aung PP, et al. (2010) Effectiveness of five artemisinin combination regimens with or without primaquine in uncomplicated falciparum malaria: an open-label randomised trial. The Lancet infectious diseases 10: 673–681.

7. Sirima SB, Ogutu B, Lusingu JPA, Mtoro A, Mrango Z, Ouedraogo A, et al. Comparison of artesunate-mefloquine and artemether-lumefantrine fixed-dose combinations for treatment of uncomplicated *Plasmodium falciparum* malaria in children younger than 5 years in sub-Saharan Africa: a randomised, multicentre, phase 4 trial. Lancet Infect Dis. 2016 ;16(10):1123-1133.

8. Myint MK, Rasmussen C, Thi A, Bustos D, Ringwald P, Lin K. Therapeutic efficacy and artemisinin resistance in northern Myanmar: evidence from in vivo and molecular marker studies. Malar J. 2017;16:143

9. Tarning J, Ashley EA, Lindegardh N, et al. Population pharmacokinetics of piperaquine after two different treatment regimens with dihydroartemisinin-piperaquine in patients with *Plasmodium falciparum* malaria in Thailand. Antimicrob Agents Chemother 2008; 52:1052–61.

10. Tjitra E, Hasugian AR, Siswantoro H, Prasetyorini B, Ekowatiningsih R, Yusnita EA, et al. Efficacy and safety of artemisinin-naphthoquine versus dihydroartemisinin-piperaquine in adult patients with uncomplicated malaria: a multi-centre study in Indonesia. Malar J. 2012 ;11(1):153.

11. Kyaw MP, Nyunt MH, Chit K, Aye MM, Aye KH, et al. (2013) Reduced Susceptibility of *Plasmodium falciparum* to Artesunate in Southern Myanmar. PLoS ONE 8(3): e57689.

12. Dondorp A, Nosten F, Stepniewska K, Day N, White N (2005) Artesunate versus quinine for treatment of severe falciparum malaria: a randomised trial. Lancet 366: 717–725

13. Wattanakoon Y, Chittamas S, Pornkulprasit V, Kanda T, Thimasarn K, Rojanawatsirivej C, et al. Six-years monitoring the efficacy of the combination of artesunate and mefloquine for the treatment of uncomplicated falciparum malaria. Southeast Asian J Trop Med Public Health. 2003, 34: 542-545.

14. Ghimire P, Rijal KR, Kafle C, Karki BS, Singh N, Ortega L, et al. Efficacy of artemether-lumefantrine for the treatment of uncomplicated *Plasmodium falciparum* malaria in Nepal. Tropical diseases, travel medicine and vaccines. 2018 ;4(1):9

15. Wijeyaratne P, Chand P, Valecha N, Shahi B, Adak T, Ansari M, et al. Therapeutic efficacy of antimalarial drugs along the eastern indo-Nepal border: a cross-border collaborative study. Trans R Soc Trop Med Hyg. 2005;99:423–9

16. Haque R, Thriemer K, Wang Z, Sato K, Wagatsuma Y, Salam MA, et al. Therapeutic efficacy of artemether-lumefantrine for the treatment of uncomplicated *Plasmodium falciparum* malaria in Bangladesh. Am J Trop Med Hyg. 2007;76:39–41

17. Phong NC, Chavchich M, Quang HH, San NN, Birrell GW, Chuang I, et al.Susceptibility of *Plasmodium falciparum* to artemisinins and *Plasmodium vivax* to chloroquine in Phuoc Chien Commune, Ninh Thuan Province, south-central Vietnam. Malar J. 2019;18(1):10.
