# Supplementary material for: Efficacy of antimalarial drugs for treatment of uncomplicated falciparum malaria in Asian region: A network meta-analysis
Source: PLoS One. 2019 Dec 19;14(12):e0225882. doi: 10.1371/journal.pone.0225882 (PMC6922314; doi:10.1371/journal.pone.0225882)
Supplement: S4 Table — (PDF) [file pone.0225882.s004.pdf]

S4 Table. Distribution of studies and comparisons

Study, year of publication	Ref. No.	Drug A	Drug B	d1	n1	d2	n2
Ashley,2005	24	DHP	DHP4	170	170	163	163
Ashley,2005	24	DHP	ASMQ	170	170	164	166
Ashley,2005	24	DHP4	ASMQ	163	163	164	166
Ashley,2004	23	DHP	DHPAS	58	67	59	67
Ashley,2004	23	DHP	ASMQ	58	67	59	67
Ashley,2004	23	DHPAS	ASMQ	59	67	59	67
Song,2011	30	DHP	AL	54	55	42	55
Song,2011	30	DHP	AMPQ	54	55	97	110
Song,2011	30	AL	AMPQ	42	55	97	110
van Vgt, 2000	37	AL	AMQ	146	150	50	50
Valecha, 2010	36	DHP	ASMQ	719	767	350	381
Tjitra,2001	34	ASSP	SP	43	53	39	52
Smithuis,2006	29	DHP	ASMQ	153	154	157	157
Smithuis,2006	29	DHP	DHPh	153	154	164	168
Smithuis,2006	29	DHP	ASMQh	153	154	159	159

Smithuis,2006	29	ASMQ	DHPh	157	157	164	168
Smithuis,2006	29	ASMQ	ASMQh	157	157	159	159
Smithuis,2006	29	DHPh	ASMQ	164	168	157	157
Smithuis,2006	29	DHPh	ASMQh	164	168	159	159
Thanh,2009	31	DHP	ASAQ	49	49	46	49
Thanh,2009	31	DHP	DHP2	49	49	55	55
Thanh,2012	32	ASAQ	DHP2	46	49	55	55
Thapa,2007	33	AL	SP	66	66	29	33
Silachamroon,2005	28	ASMQ	ASMQ2	59	59	57	58
Huong, 2003	27	ASSP	ASCQ	48	62	39	61
Trung,2009	35	DHP	DHP2	51	51	52	52
Kshirsagar,2000	25	AL	CQSP	62	65	15	76
Lefevre,2001	26	AL	ASMQ	164	257	53	54
Rachmawati,2010	14	AL	ASSP	23	23	24	24
Wilairatana,2002	38	DHP	ASMQ	189	234	80	118

Drug A, Drug B: drugs used in a pair comparison; d1/d2: number of patients with absence of parasitemia in the arm of drug A/drug B; n1/n2: total number of patients in the arm of drug A/ drug B; The list of drugs shares the same legend with Appendix Table #.
