# Supplementary material for: Efficacy of antimalarial drugs for treatment of uncomplicated falciparum malaria in Asian region: A network meta-analysis
Source: PLoS One. 2019 Dec 19;14(12):e0225882. doi: 10.1371/journal.pone.0225882 (PMC6922314; doi:10.1371/journal.pone.0225882)
Supplement: S5 Table — (PDF) [file pone.0225882.s005.pdf]

S5 Table. Risk of bias assessment by the review authors

	Author, Year of publication	Randomization	Allocation concealment	Blinding	Sample size Calculation [1]	Types of analysis
1	Rachmawati, 2010 [14]	Low: 3:1 randomization	Unclear: not stated	Unclear: not stated	No	NA
2	Ashley, 2004 [23]	Low: computer generated randomisation in blocks	Low: sealed envelopes	Low: blinded (investigator & lab staff	No	ITT
3	Ashley, 2005 [24]	Low: computer generated randomisation in blocks	Low: sealed envelopes	Low: blinded (lab staff)	No	modified ITT
4	Kshirsaga, 2000 [25]	Low: computer-generated randomization in blocks scheme	Low: Seems central randomization	Low: double blinded	No	ITT
5	Lefevre, 2001 [26]	Unclear: randomised, no further details	Unclear: not stated	High: open label	No	ITT
6	Nguyen, 2003 [27]	Unclear: patients were randomized, no further details	Unclear: not stated	Unclear: not stated	No	NA
7	Silachamroon, 2005 [28]	Unclear: randomly allocated	Unclear: sequentially assigned	High: open label	No	ITT
8	Smithuis, 2006 [29]	Unclear: randomly allocated	Low: unmarked & sealed opaque envelopes	High: open-label	Yes	non-inferiority
9	Song,2011 [30]	Low: pre-generated	Low: sealed, opaque	High: open label	Yes	NA

		randomization list (20 blocks	envelopes			
10	Thanh,2009 [31]	Unclear: randomly allocated	Unclear: sequentially assigned	High: open label	No	NA
11	Thanh,2012 [32]	Unclear: randomly allocated	Unclear: sequentially assigned	High: open label	NA	PP
12	Thapa,2007 [33]	Low: 2:1 randomization	Unclear: not stated	High: open label	Yes	NA
13	Tjitra,2001 [34]	Low: phase III, randomized	Low: sealed envelopes	High: open label	Yes	NA
14	Trung,2009 [35]	Low: block randomization	Unclear: not stated	Unclear: not stated	NA	NA
15	Valecha,2010 [36]	Low: phase III, computer generated randomisation	Low: sealed envelopes	Low: open label (blinded outcome assessment)	No	ITT
16	van Vgt,2000 [37]	Low: 3: 1 randomization	Low: sealed envelopes	High: open label	Yes	ITT
17	Wilairatana,2002 [38]	Low: 2:1 randomization	Unclear: not stated	High: open label	No	NA

1: Whether sample size calculation was described (Yes) or not (No); ITT: intention-to-treat analysis; RCT: Randomised-clinical trial
