# Supplementary material for: Efficacy of antimalarial drugs for treatment of uncomplicated falciparum malaria in Asian region: A network meta-analysis
Source: PLoS One. 2019 Dec 19;14(12):e0225882. doi: 10.1371/journal.pone.0225882 (PMC6922314; doi:10.1371/journal.pone.0225882)
Supplement: S6 Table — (PDF) [file pone.0225882.s006.pdf]

S6 Table. Treatment relative ranking

Interventions	Abbreviation	SUCRA (%)	PrBest	Mean Rank
Artemether- lumefantrine	AL	73.5	0.0	4.4
Artemisinin-piperaquine	AMPQ	57.7	0.0	6.5
Artesunate-amodiaquine	ASAQ	52.5	0.2	7.2
Artesunate-chloroquine	ASCQ	75.4	3.9	4.2
Artesunate mefloquine	ASMQ	22.0	0.0	11.1
Artesunate mefloquine home treatment (not supervised)	ASMQh	46.0	0.0	8.0
Artesunate mefloquine (2-day course)	ASMQ2	13.6	0.0	12.2
Artesunate plus sulfadoxine-pyrimethamine	ASSP	62.8	0.1	5.8 |
Dihydroartemisinin-piperaquin	DHP	99.2	92.4	1.1
Chloroquine	CQ	25.0	0.0	10.7
Dihydroartemisinin piperaquine(home treatment/not supervised)	DHPh	30.5	0.1	10.0
Dihydroartemisinin piperaquine 4-day course	DHP4	22.0	0.0	11.0
Dihydroartemisinin piperaquine & added artesunate	DHPAS	45.6	0.0	8.1
Sulfadoxine-pyrimethamine	SP	74.1	3.3	4.4
