# Supplementary material for: Efficacy of antimalarial drugs for treatment of uncomplicated falciparum malaria in Asian region: A network meta-analysis
Source: PLoS One. 2019 Dec 19;14(12):e0225882. doi: 10.1371/journal.pone.0225882 (PMC6922314; doi:10.1371/journal.pone.0225882)
Supplement: S1 Fig — (PDF) [file pone.0225882.s007.pdf]

S1 Fig. Forest plot of direct pairwise comparison of antimalarial regimens

A) Estimates of individual drug comparisons

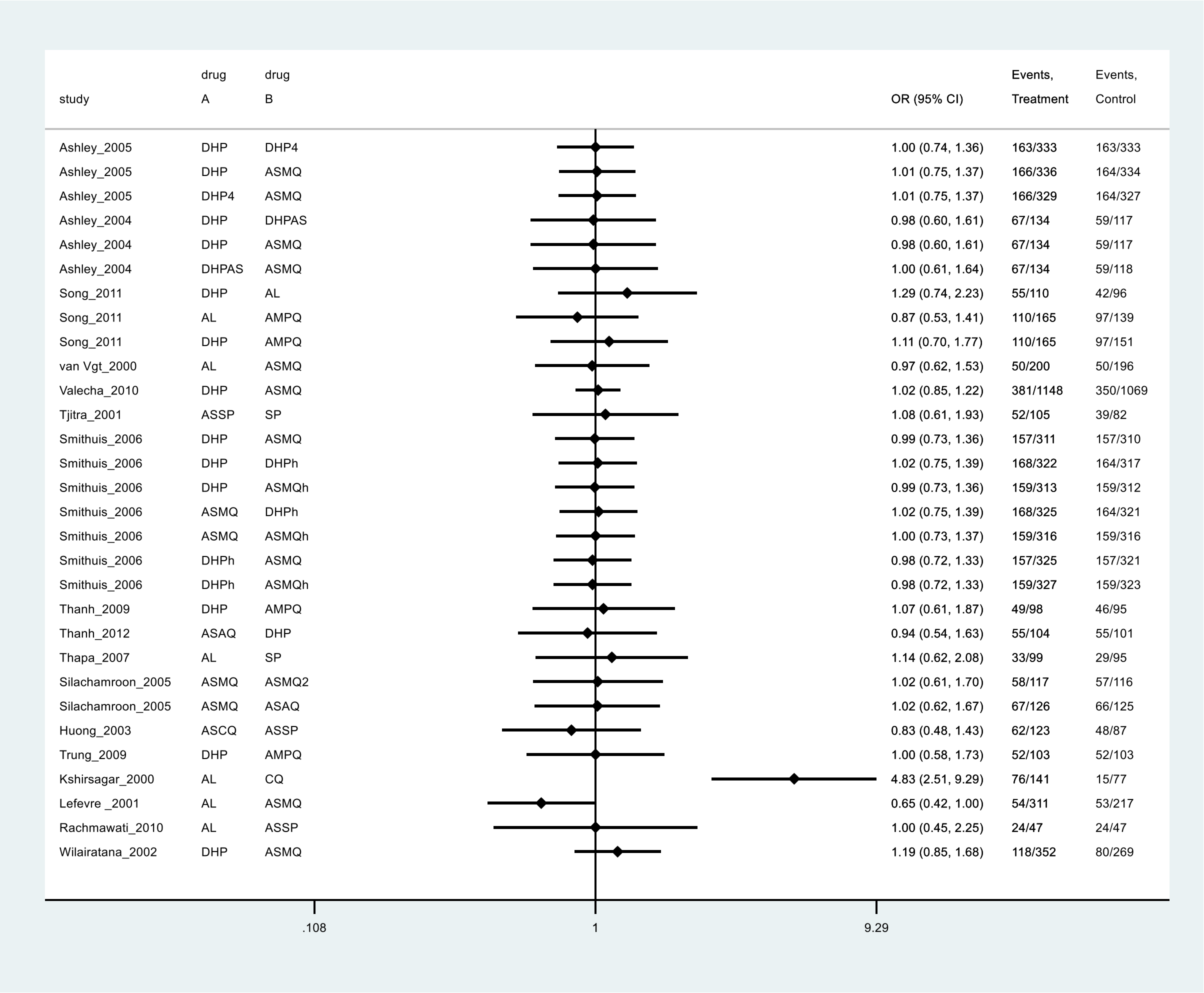

B) Individual trials in group of drug

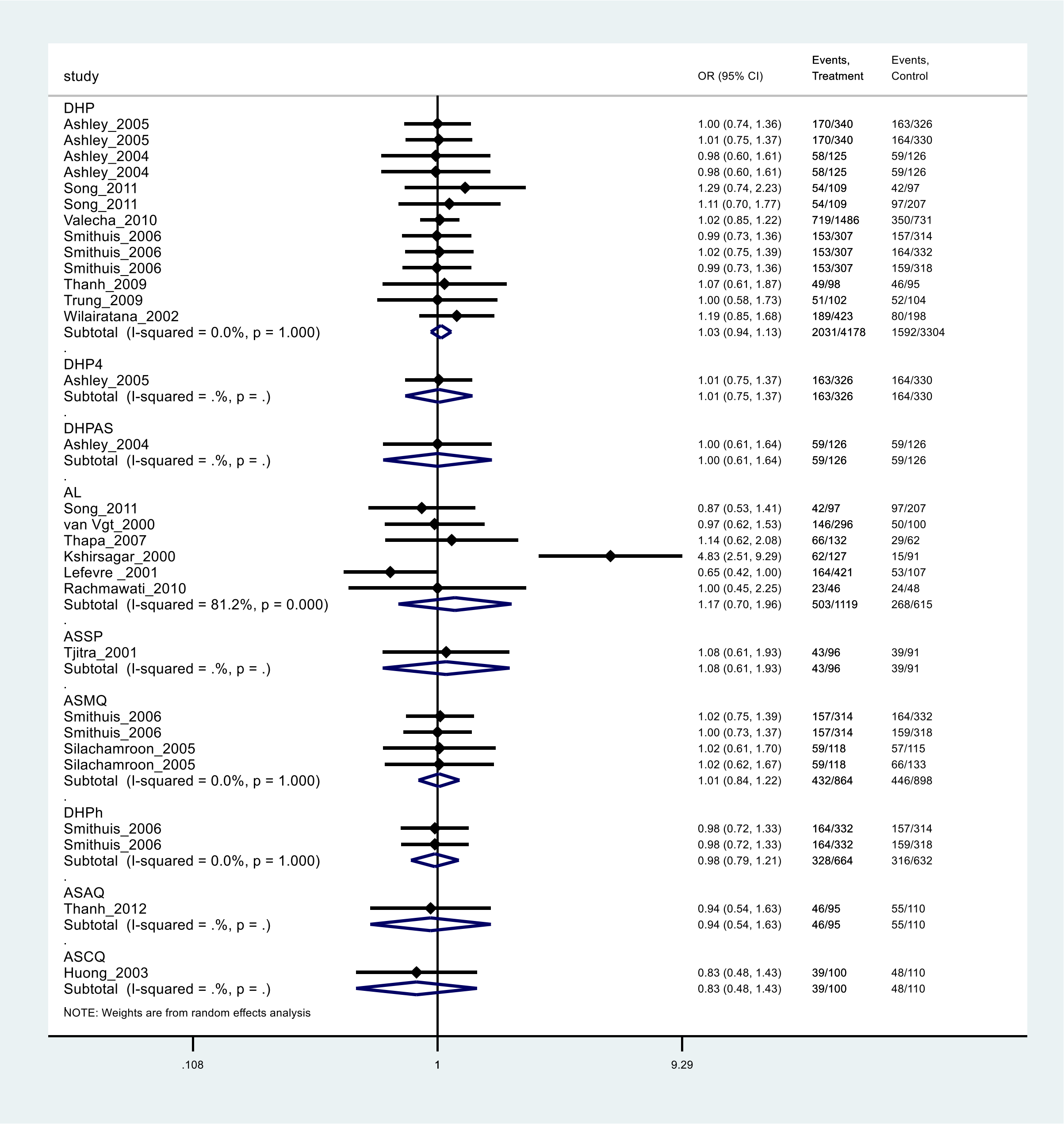

Abbreviations of the drugs are as stated in Fig 3
