# Supplementary material for: Efficacy of antimalarial drugs for treatment of uncomplicated falciparum malaria in Asian region: A network meta-analysis
Source: PLoS One. 2019 Dec 19;14(12):e0225882. doi: 10.1371/journal.pone.0225882 (PMC6922314; doi:10.1371/journal.pone.0225882)
Supplement: S2 Fig — (PDF) [file pone.0225882.s008.pdf]

S2 Fig. Inconsistency plot with loop-specific heterogeneity

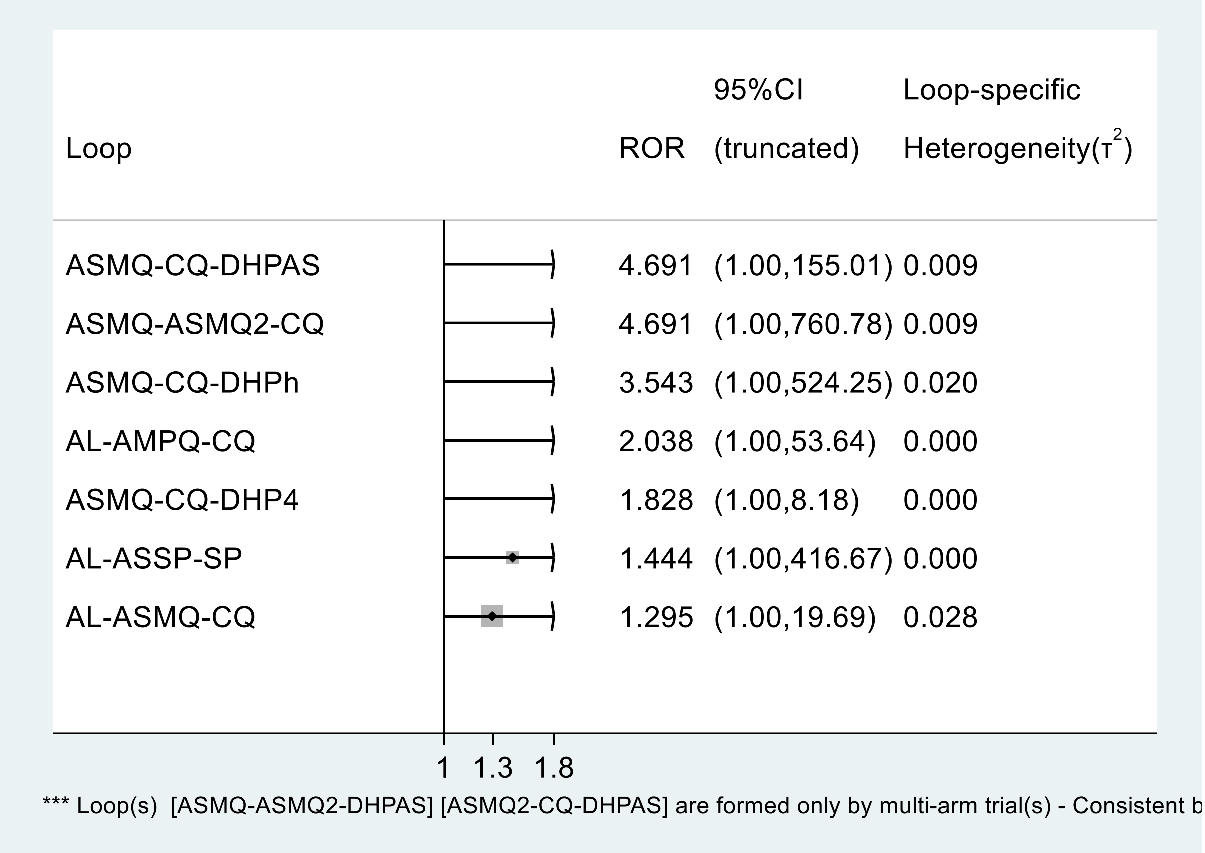

Note: ASMQ-ASMQ2-DPHPAS and ASMQ2-CQ- DHPAS are formed only by multi-arm trial(s) - Consistent by definition.

Abbreviations of the drugs are as stated in Table 1. It is showing for each loop the ratio of odds ratios between direct and indirect estimates.
AL-AMPQ-CQ has ROR of 2.038 (1-53.64), suggesting that direct estimate could not be difference from the indirect estimate or vice versa [39].
