# Supplementary material for: Efficacy of antimalarial drugs for treatment of uncomplicated falciparum malaria in Asian region: A network meta-analysis
Source: PLoS One. 2019 Dec 19;14(12):e0225882. doi: 10.1371/journal.pone.0225882 (PMC6922314; doi:10.1371/journal.pone.0225882)
Supplement: S3 Fig — (PDF) [file pone.0225882.s009.pdf]

S3 Fig. Comparison-adjusted funnel plot of the placebo-controlled antimalarial trials

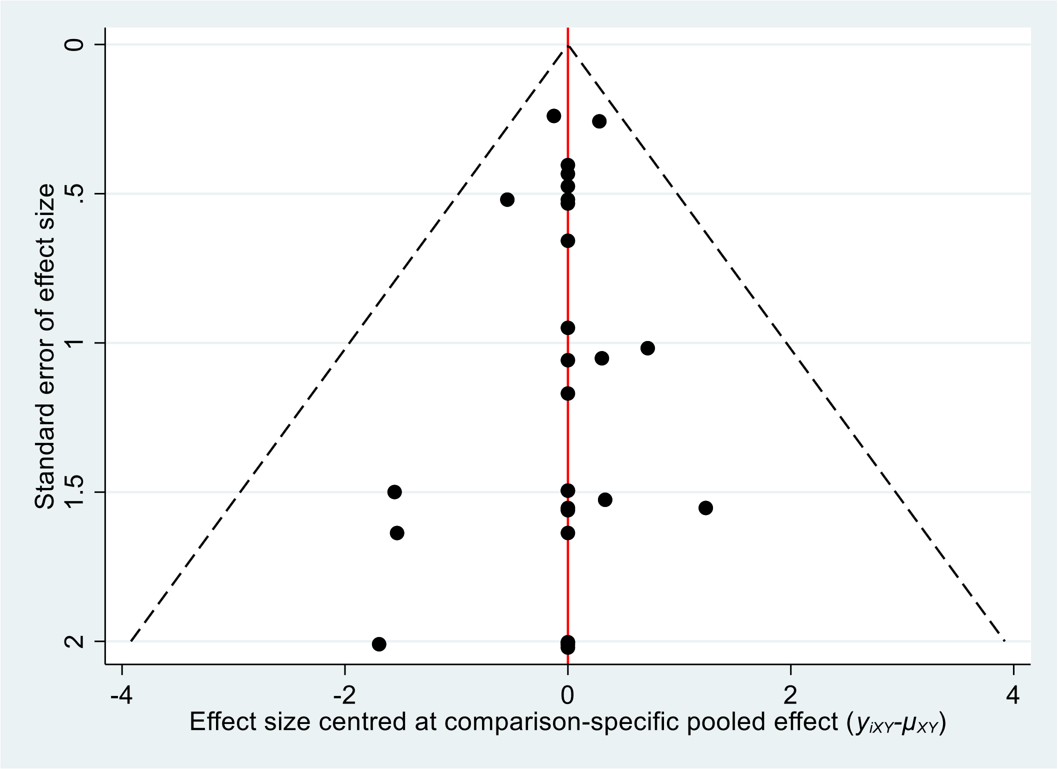

The comparison-adjusted funnel plot appears symmetric, implying the absence of small-study effects in the network
