# Supplementary material for: Efficacy of antimalarial drugs for treatment of uncomplicated falciparum malaria in Asian region: A network meta-analysis
Source: PLoS One. 2019 Dec 19;14(12):e0225882. doi: 10.1371/journal.pone.0225882 (PMC6922314; doi:10.1371/journal.pone.0225882)
Supplement: Attachment — Submitted filename: Our reply_ (12112019).doc [file pone.0225882.s011.doc]

| No | Comment | Our reply | Amendments |
| --- | --- | --- | --- |
| 1 | Suggest defining “Asian region” in Methods-Study selection  (since this definition was used to include studies in the analysis)  or the Introduction (since line 84 may suggest to some  readers that Asian region is synonymous with GMS). | We have provided an operational definition of the Asian region.  Thank you | **Methods**  **Selection of study**  An operational definition of the Asian region for this particular study covers countries in three regions of Southeast Asia, South Asia and East Asia. |
| 2 | Suggest defining uncomplicated malaria and indicating whether this definition includes patients with hyperparasitemia but without signs or symptoms of severe malaria. | According to the WHO revised criteria, *P. falciparum* parasitaemia >5% or > 200,000/μl is one of the feature of severe malaria.  In the present meta-analysis study, some studies indicated specific information on parasitemia as less than 200,000/μl. But, not all trials provided such information. All studies had confirmed P. falciparum and excluded patients with clinical features of severe malaria as described by WHO.  Hence, we selected the studies if they had indicated patients with uncomplicated malaria (excluding severe malaria) are recruited.  As suggested, we now have provided additional information for clarification.  Thank you | **Selection of study**  Uncomplicated malaria caused by the P. falciparum parasite in this study is defined as patients with the presence of *P. falciparum* and having symptoms that are non-specific and with no clinical or laboratory findings of severe organ dysfunction. |
| 3 | Line 45: Suggest assigning abbreviations for all treatments at first use here and using them consistently throughout the manuscript, tables, and figures. | We have updated the abbreviations in consistency manner throughout the manuscript. In the abstract, please allow us to use abbreviations, only if it is necessary to repeat.  Thank you |  |
| 4 | Line 57: The conclusion that DHP is superior to AL is not supported by the OR (95% CI) data in line 57 and Table 2. | Please, accept our apology for a typing mistake in the previous version. It should be described as (OR 2.5, 95%CI: 1.08 to 5.8) as shown in Table 2 pertinent to ‘Network estimate’ column. We have done the corrections in this revised version  Moreover, we have briefly added a caution over drug resistance status of the area.  Thank you for giving the valuable inputs. | **Abstract**  **Results**  In general, DHP had better efficacy than AL at day 28 (DHP vs AL: OR 2.5, 95%CI:1.08-5.8). There is low certainty evidence due to limited number of studies and small trials.  **Conclusion**  The findings suggest the superiority of DHP (3–day course) to AL and other comparator ACTs are with the overall low/very low quality of evidence judgements.  Moreover, one drug regimen is better than another is only, if current drug-resistance patterns are at play. For example, the AL might be better than DHP in areas where both artemisinin and piperaquine resistance patterns are prevalent.  **Text**  **Results**  **Fourteen-node analysis**  For instance, DHP compared to AL in a single trial and there was a superiority of DHP in cure rate at day 28 (OR 2.5, 95%CI: 1.08 to 5.8) (Table 2) |
| 5 | Lines 59, 308, 312: The suggestion to analyze safety information in future studies seems to come out of nowhere. Does it relate to the benefit vs. harm concept mentioned in line 242? In any case, suggest mentioning at least one result in the Abstract to support this statement. | We gratefully agreed on the inputs given.  We have recommended future studies to included safety analysis so as to cover the benefit versus harm concept.  Thank you | **Abstract**  **Conclusions**  In the light of benefit versus harm concept, future analysis including safety information are recommended.  **Discussion**  **Implications**  Moreover, in the light of benefit versus harm concept, a NMA of the relative safety of different antimalarial treatment is needed.  **Conclusions**  In the light of benefit versus harm concept, future analysis with safety information is recommended. |
| 6 | Line 100: Suggest providing 1-2 examples of conflicting results in other studies that the present study was designed to address. | We have added salient examples in brief.  Thank you | **Text**  **Introduction**  For example, a review by Zani and associates reported that in Africa, there was better efficacy in DHP than AL at day 28. However, such relationship was not shown in Asia [7]. This reflects that efficacy of antimalarial is related to whether the area is with artemisinin-sensitive parasite populations or not [6,9]. |
| 7 | Line 108: This sentence suggests that ASCQ (mentioned in the Abstract) is currently used in Asian region. Is this true? If so, where? How is “currently” defined? | Our main focus was the currently used 5 ACTs including DHP, AL. These currently used drugs compared with other antimalarials. All comparators are not currently used antimalarials (e.g ASCQ in this case).  We have indicated the comparator drugs as available in the trials.  For clarity, we have removed this result from abstract.  To be more specific, we have added a concern over an issue of drug resistance for an interpretation of the results. We very much appreciated the points provided.  Thank you | **Abstract**  **Results**  In general, DHP had better efficacy than AL at day 28 (DHP vs AL: OR 2.5, 95%CI:1.08-5.8).There is low certainty evidence due to limited number of studies and small trials.  **Conclusions**  The findings suggest the superiority of DHP (3–day course) to AL and other comparator ACTs are with the overall low/very low quality of evidence judgements. Moreover, one drug regimen is better than another is only, if current drug-resistance patterns are at play. For example, the AL might be better than DHP in areas where both artemisinin and piperaquine resistance patterns are prevalent. |
| 8 | Line 154: Does “preferred” mean that the ITT population analysis was used over another analysis whenever it was available, or that only ITT population analyses were used? | For clarity, we have updated this information.  Thank you for the valuable input. | **Data synthesis**  We preferred the intention-to-treat (ITT) analysis over another analysis whenever it was available. |
| 9 | Line 191: Suggest indicating that 5043 refers to total number of patients. | We have clarified this information.  Thank you | **Results**  After removal of duplicates and screening of titles and abstracts, 34 full-text papers were evaluated and 17 studies, incorporating 5043 total number of patients were finally included in this review [14, 23-38]. |
| 10 | Line 197: Suggest stating the range of % males as well. | We have updated this information. Thank you | The majority of participants in the studies included were males (range from 51% to 96%) with mean age between 5.9 to 29 years. |
| 11 | Line 302: This statement seems misleading considering that SP and CQ are almost completely ineffective in Southeast Asia due to drug resistance. Suggest revising. | We have clarified the statement.  Thank you | **Implications**  There is evidence that DHP has better treatment outcome than monotherapy such as SP and CQ, which are almost completely ineffective in Southeast Asia due to drug resistance. |
| 12 | In general, the authors should state the caveat that one drug regimen is better than another only if current drug-resistance patterns are at play. For example, AL may be far superior to DHP in areas where both artemisinin and piperaquine resistance are prevalent. | Much appreciated for giving the valuable inputs.  We have updated the conclusion, taking the points provided.  Thanking you | **Abstract**  **Conclusions**  Moreover, one drug regimen is better than another is only if current drug-resistance patterns are at play. For example, the AL might be better than DHP in areas where both artemisinin and piperaquine resistance patterns are prevalent.  **Text**  **Conclusions**  The findings suggest the superiority of DHP to AL and other comparator ACTs are with the overall low/very low quality of evidence judgements. Moreover, one drug regimen is better than another is only, if current drug-resistance patterns are at play. For example, the AL might be better than DHP in areas where both artemisinin and piperaquine resistance patterns are prevalent [7,9]. |
| 13 | Table 1: Funding for Studies 1, 12 and 16 are missing. If information is not available, suggest stating so. | Thank you for pointing out the missing information. We have updated the Table 1 accordingly.  Thank you | **Table 1** |
|  | **Journal requirements:** |  |  |
| 1 | When submitting your revision, we need you to address these additional requirements.   Please ensure that your manuscript meets PLOS ONE's style requirements, including those for file naming. The PLOS ONE style templates can be found at  <http://www.journals.plos.org/plosone/s/file?id=wjVg/PLOSOne_formatting_sample_main_body.pdf> and [http://www.journals.plos.org/plosone/s/file?id=ba62/PLOSOne_formatting_sample_title_authors_affiliations.pd](http://www.journals.plos.org/plosone/s/file?id=ba62/PLOSOne_formatting_sample_title_authors_affiliations.pdf) | We have followed the *PLOS One* format.  Thank you |  |
| 2 | We noticed you have some minor occurrence of overlapping text with the following previous publication(s), which needs to be addressed:  <https://apps.who.int/iris/bitstream/handle/10665/275867/9789241565653-eng.pdf?ua=1> <https://malariajournal.biomedcentral.com/articles/10.1186/1475-2875-11-153> [http://www.ajtmh.org/content/journals/10.4269/ajtmh.2006.74.991#html_fulltext](http://www.ajtmh.org/content/journals/10.4269/ajtmh.2006.74.991#html_fulltext) <https://bmcinfectdis.biomedcentral.com/articles/10.1186/s12879-018-3506-x> <https://aac.asm.org/content/aac/47/3/901.full.pdf>  In your revision ensure you cite all your sources (including your own works), and quote or rephrase any duplicated text outside the methods section. Further consideration is dependent on these concerns being addressed. | We have updated the whole text and improved any duplications.  We have checked % similarity and it shows 20% similarity (attached under “Other”). We sincerely feel that this is an acceptable limit for a meta- analysis study.  Thank you |  |
| 3 | Thank you for stating the following financial disclosure: “none".   a. Please provide an amended Funding Statement that declares *all* the funding or sources of support received during this specific study (whether external or internal to your organization) as detailed online in our guide for authors at <http://journals.plos.org/plosone/s/submit-now>. b. Please state what role the funders took in the study.  If any authors received a salary from any of your funders, please state which authors and which funder. If the funders had no role, please state: "The funders had no role in study design, data collection and analysis, decision to publish, or preparation of the manuscript."    * Please include your amended statements within your cover letter; we will change the online submission form on your behalf. | We have added this information in PRISMA checklist and the cover letter.  Thank you | **Cover letter**  The authors received no specific funding for this work. |
| 4 | Please include your tables as part of your main manuscript and remove the individual files. Please note that supplementary tables (should remain/ be uploaded) as separate "supporting information" files | We have placed Tables in the manuscript. Thank you |  |
